# Treating symptomatic uterine fibroids with myomectomy: current practice and views of UK consultants

**DOI:** 10.1186/s10397-017-1033-1

**Published:** 2017-12-19

**Authors:** R. Mallick, F. Odejinmi

**Affiliations:** Department of Gynaecology, Barts Health NHS Trust, Whipps Cross University Hospital, London, E11 1NR UK

Sir,

We read with great interest this potentially landmark survey by Sirkeci and colleagues investigating the attitudes of UK obstetricians and gynaecologists to the treatment of uterine fibroids [1].

Such surveys, as the authors acknowledge, are plagued by low response rates; however, they can also be subject to significant recall bias and furthermore those who respond to such questionnaires tend to have an interest in the subject matter. It is therefore surprising to find that a third of those who responded would still not set a limit to the number of fibroids removed hysteroscopically. There is increasing evidence that submucous fibroids greater than 4 cm, especially when there are multiple types of fibroids present, should be removed laparoscopically as hysteroscopic resection does not allow complete removal and increases the risk of recurrence [2]. Furthermore, only 43% of respondents who perform open myomectomies would aim to remove all fibroids, resulting in an increased risk of recurrence and the need for secondary intervention, which is known to be significantly less successful than primary interventions particularly with regard to fertility and symptom resolution [3].

Most of the respondents agreed that the more complex operations should be performed by those with more experience; however, conversely, those with more experience were least likely to perform a laparoscopic myomectomy, which in 2017 should be considered the “gold standard” surgical treatment.

It is good to see that the numbers of gynaecologists in the UK performing myomectomies have increased since the previous survey over a decade ago; however, these numbers still remain small. Furthermore, disappointingly, almost 40% of women still do have access to facilities offering uterine artery embolisation.

With regard to perimenopausal women, the number of respondents who would perform myomectomy in this cohort has increased. However, a large proportion of women are still deprived of this uterine sparing option by a considerable number of gynaecologists despite existing evidence to show that myomectomy is as safe in the older woman once the issues surrounding leiomyosarcoma are investigated and discussed [4]. Although myomectomy would not be the first-line treatment of choice for women in this age group, many patients do wish to conserve their uterus and they should be given adequate information and counselling to allow them to make an informed choice.

Finally, with the changing face of the gynaecological workforce in the UK as reiterated by this survey, though the authors advise caution in the interpretation of the results, it sends a clear message of disparity in the availability of the various different treatment modalities for fibroids. It is thus important that we re-evaluate the management options for such women and perhaps consider setting up specialised tertiary referral centres to allow all women access to experienced surgeons who would take a holistic multidisciplinary approach to management and have access to all the various treatment modalities in line with what is readily available to women with endometriosis via the “BSGE badged” endometriosis centres.
